# Identification of prognostic collagen signatures and potential therapeutic stromal targets in canine mammary gland carcinoma

**DOI:** 10.1371/journal.pone.0180448

**Published:** 2017-07-06

**Authors:** Ashley Case, Becky K. Brisson, Amy C. Durham, Suzanne Rosen, James Monslow, Elizabeth Buza, Pascale Salah, Julie Gillem, Gordon Ruthel, Sridhar Veluvolu, Veronica Kristiansen, Ellen Puré, Dorothy C. Brown, Karin U. Sørenmo, Susan W. Volk

**Affiliations:** 1 Department of Clinical Sciences and Advanced Medicine, School of Veterinary Medicine, University of Pennsylvania, Philadelphia, PA, United States of America; 2 Department of Pathobiology, School of Veterinary Medicine, University of Pennsylvania, Philadelphia, PA, United States of America; 3 Department of Biomedical Sciences, School of Veterinary Medicine, University of Pennsylvania, Philadelphia, PA, United States of America; 4 Department of Companion Animal Clinical Sciences, Veterinary Medicine and Biosciences, Norwegian University of Life Sciences, Oslo, Norway; Colorado State University, UNITED STATES

## Abstract

Increasing evidence indicates that the tumor microenvironment plays a critical role in regulating the biologic behavior of breast cancer. In veterinary oncology, there is a need for improved prognostic markers to accurately identify dogs at risk for local and distant (metastatic) recurrence of mammary gland carcinoma and therefore would benefit from adjuvant therapy. Collagen density and fiber organization have been shown to regulate tumor progression in both mouse and human mammary tumors, with certain collagen signatures predicting poor outcomes in women with breast cancer. We hypothesized that collagen signatures in canine mammary tumor biopsies can serve as prognostic biomarkers and potential targets for treatment. We used second harmonic generation imaging to evaluate fibrillar collagen density, the presence of a tumor-stromal boundary, tumor associated collagen signatures (TACS) and individual collagen fiber characteristics (width, length and straightness) in grade I/II and grade III canine mammary tumors. Collagen density, as well as fiber width, length and straightness, were inversely correlated with patient overall survival time. Notably, grade III cases were less likely to have a tumor-stromal boundary and the lack of a boundary predicted poor outcome. Importantly, a lack of a defined tumor-stromal boundary and an increased collagen fiber width were associated with decreased survival even when tumor grade, patient stage, ovariohysterectomy status at the time of mammary tumor excision, and histologic evidence of lymphovascular invasion were considered in a multivariable model, indicating that these parameters could augment current methods to identify patients at high risk for local or metastatic progression/recurrence. Furthermore, these data, which identify for the first time, prognostic collagen biomarkers in naturally occurring mammary gland neoplasia in the dog, support the use of the dog as a translational model for tumor-stromal interactions in breast cancer.

## Introduction

In spite of major advances in diagnosis and treatment over the past several decades, breast cancer remains a major cause of morbidity and premature death for both human and veterinary patients throughout the world. Breast cancer is the most frequently diagnosed cancer in women and sexually intact female dogs [1–6]. Synergy between veterinarians, physicians, and other scientific health and environmental professionals has been promoted in an initiative known as “One Health” to improve the lives of all species through the integration of human and veterinary medical research [7]. This “One Health” approach may more efficiently and simultaneously transform breast cancer treatment in women and female dogs. This approach applies not only novel diagnostics and therapeutics to veterinary oncologic patients from cutting-edge human breast cancer medical strategies, but also informs human medicine through the unique advantages of the spontaneous, naturally occurring canine breast cancer model.

Approximately half of all canine mammary gland tumors are malignant, and depending on subtype, stage, and grade can be associated with a significant risk of local and distant (metastatic) recurrence [2–6, 8]. Similar to that which occurs in their human counterparts, there are two major obstacles limiting successful outcomes in dogs with mammary carcinoma: 1) accurate identification of dogs at risk for recurrence and 2) effective therapies for these at-risk individuals. The lack of accurate prognostic indicators results in increased morbidity and mortality due to both over-treatment of patients bearing malignant tumors with low metastatic potential and inadequate therapies for those requiring early, aggressive intervention.

At present, histopathologic grade represents one of the most important prognostic indicators and is commonly incorporated in the decision to determine if systemic therapy is warranted in dogs with mammary tumors. This three-tiered grading system, that incorporates tubule formation, nuclear pleomorphism, and mitotic index [9, 10], is based on the Elston and Ellis grading system for human breast cancer [11, 12]. These grading systems successfully predict prognosis for the majority of individuals with breast cancer, as women with grade I breast cancer have significantly longer survival times than those with grade II and III breast cancer [12]. Similarly, dogs with grade III mammary carcinomas have a 21-fold increased risk of death compared to dogs with grade I and II tumors [11]. In addition, lower stage (which incorporates a smaller primary tumor size and absence of regional lymph node and distant metastasis), the absence of lymphovascular invasion and the performance of ovariohysterectomy (OHE) with mammary tumor resection have been shown to predict improved overall survival (OS) [13–16]. Nevertheless, similar to their human counterparts, there are a significant number of canine patients whose outcome is not predicted with current clinical and histopathologic indicators [10, 11, 13]. Additional criteria appear necessary to both optimize and accurately prognosticate this sub-group and to refine predictive information for subpopulations of patients with high-, intermediate- and low-grade tumors.

Numerous studies suggest that collagen in the tumor microenvironment (TME) plays a critical role in regulating the growth and spread of cancer through its ability to provide physical, biochemical, and biomechanical cues to both tumor and non-tumor cells [17–21]. In fact, increased mammographic density, which correlates to the abundance of collagen-rich fibroglandular tissue, is one of the best known risk factors for breast cancer development [22]. More recent data reveal that specific tumor-associated collagen signatures (TACS) can predict recurrence in women and murine models [20, 21, 23–25] and highlight the importance of collagen organization in determining whether collagen plays a tumor-permissive or tumor-restrictive role in breast cancer progression. Given this critical role for collagen in modulating breast cancer development and progression, it is not surprising that recent studies suggest that targeting the tumor stroma is a potential therapeutic strategy for tumor control [26–31]. Furthermore, the identification and/or confirmation of tumor-permissive or tumor-restrictive stromal signatures are key to developing similar therapies in the dog.

We hypothesize that collagen signatures identified in standard formalin-fixed canine mammary tumor biopsy specimens can serve as prognostic biomarkers to distinguish dogs at high-risk for poor clinical outcomes whom require adjuvant therapies, from low-risk patients who do not. Here, we present evidence that collagen plays an important role in modulating the biologic behavior of canine mammary gland tumors and show that specific collagen signatures defined by second harmonic generation (SHG) imaging are predictive of aggressive biological behavior and poor outcomes. We demonstrate that a lack of a defined tumor-stromal boundary and collagen width predict OS independently of tumor grade, patient stage, OHE status, and lymphovascular invasion at the time of mammary tumor excision. As spontaneously occurring canine mammary gland carcinoma has been proposed to have advantages as a model of human breast cancer compared to murine models [32–34], together with an increasing awareness of the critical role that the stromal TME plays in breast cancer biology and therapeutics, defining tumor-permissive collagen features in the dog has important implications for a One Health approach to breast cancer.

## Materials and methods

### Case selection

Canine mammary gland carcinoma biopsy samples were obtained from the University of Pennsylvania School of Veterinary Medicine and the School of Veterinary Science, Norwegian University of Life Sciences (NULS; Oslo, Norway) to determine the association between collagen patterns and outcome. Clinical cases from the University of Pennsylvania were selected from dogs participating in an on-going prospective study by the Penn Vet Shelter Canine Mammary Tumor Program (PVSCMTP) or from the Biopsy Service Archives of the Penn Vet Diagnostic Laboratory (PVDL). Norwegian biopsy samples and outcome data were obtained from a completed prospective clinical trial examining clinical outcome in client-owned dogs with spontaneous mammary carcinoma [13]. These studies were approved by the University of Pennsylvania Institutional Animal Care and Use Committee (# 804298 to KUS) or by the Institutional Animal Care and Use Board at the Norwegian School of Veterinary Science [13]. For dogs presenting with multiple primary mammary tumors at the time of diagnosis, the biopsy sample from the highest grade tumor was selected for analysis. To avoid inter-observer variability in histological interpretation that has been documented in recent studies [35, 36], all biopsies were reviewed by a single board-certified veterinary pathologist (ACD). Tumors were diagnosed as grade I (n = 13), grade II (n = 2), or grade III (n = 19) mammary carcinomas.

### Clinical and outcome data

Staging was based upon primary tumor size and documented regional or distant metastases (stages 1–5) according to the modified staging system [37]. The dogs included in the PVSCMTP and the NULS cases were all staged prior to surgery. Specifically, all dogs had tumor measurements, complete blood counts, serum chemistry profiles and three-view thoracic radiographs. Lymph nodes were evaluated by clinical examination (palpation) or fine needle aspirate with cytological examination and excisional biopsy performed if abnormal according to the clinical exam or positive cytological evaluation. In addition, lymph nodes were removed during tumor resection if the tumor was located in the 5^th^ mammary gland and a regional or chain mastectomy including the 5^th^ gland was performed. Dogs with clinically normal lymph nodes but no confirming biopsies were classified as negative; whereas dogs with positive lymph nodes were classified as having at least stage 4 disease. Dogs selected from the PVDL biopsy service archives were staged based on information provided by the submitting veterinarians collected through medical records. Follow-up questionnaires and phone calls provided additional information on tumor size, documented regional and distant metastatic disease, OHE status and timing with respect to surgery, and the use of adjuvant chemotherapy. Dogs in the two prospective trials were monitored regularly for recurrence or metastasis (every 4–6 months) for the rest of their lives. Dogs treated through their local veterinarians were not monitored on a schedule but rather as needed according to clinical signs.

Based on the available staging data, 25 dogs did not have documented metastatic disease at the time of diagnosis (stage ≤3), while nine dogs had evidence of regional lymph node (N = 7) or distant (N = 2) metastases (stage 4 and 5, respectively). Specifically, eleven dogs had stage 1, two dogs had stage 2, and nine dogs had stage 3 disease. Primary tumor size was unavailable for three dogs. These dogs were classified as having stage ≤3 as they did not have documented metastases at diagnosis. The two dogs with stage 5 disease were not included in clinical outcome analyses. Ten dogs were spayed concomitantly with surgery; the other dogs were spayed prior to tumor development or remained intact. Only one dog (PVSCMTP 2; S1 Table) received adjuvant chemotherapy (doxorubicin, 5-flurouracil, cyclophosphamide).

Histopathologic evidence of lymphovascular invasion and completeness of surgical excision was recorded. Of the 34 biopsy samples utilized in this study, we were unable to obtain clinical outcome data on 5 of these patients (S1 Table). These five samples were included to compare collagen signatures between grade I/II and grade III tumors, but were not used for the subsequent analysis of collagen signatures on clinical outcome. A total of fourteen dogs (11 of 29 dogs with available outcome data) had evidence of lymphovascular invasion on histopathology. None of the grade I/II biopsies had evidence of lymphovascular invasion, while 14/19 grade III biopsies had documented lymphovascular invasion (11/15 from dogs with available outcome data). Nine histopathological samples (grade I tumor (N = 1); grade III tumors (N = 8)) were noted to have tumor cells extending to the surgical margins (incomplete resection).

OS was defined as time from date of diagnosis (date of the biopsy) to date of death due to any cause. Dogs lost to follow-up or still alive were censored at the time of last known status in the survival analysis. Outcome data up to January 2017 was used. Censored dogs had a median follow-up time of 650 days (range: 18–944 days). Disease-free survival (DFS) was defined as time from date of diagnosis to the date of any tumor-related event (including development of lymph node, pulmonary, or other distant metastasis, grossly evident local recurrence or the development of new mammary tumors). Dogs that were alive or lost to follow-up without recurrence or died with no evidence of a tumor-related event were censored in the DFS analysis. Censored dogs (12/27 stage 1–4 with outcome data) had a median follow-up time of 542 days (range: 4–944 days).

### SHG image acquisition

All tissues were formalin-fixed and paraffin embedded, as previously described [13]. Imaging of fibrillar collagen was performed on a Leica SP5 confocal/multiphoton microscope (Leica Microsystems, Inc., Mannheim, Germany) by tuning the Coherent Chameleon Ultra II Ti:Sapphire laser (Coherent Inc., Santa Clara, CA) to 800 nm and collecting SHG signal on a nondescanned detector configured to capture wavelengths <495 nm (20x objective). Five images per case were obtained from at least 2–3 non-overlapping areas of interest containing both tumor cells and stromal collagen but free of artifact identified on hematoxylin and eosin (H&E) stained histologic sections. Specifically, these regions were taken within the tumor mass, as opposed to the tumor periphery, capturing the multiple boundaries that occur throughout the tumor between islands of mammary carcinoma cells and local stroma. SHG images were obtained from these areas of interest on corresponding unstained histologic sections for each tumor sample. To distinguish true SHG signal from autofluorescence, fluorescence images at wavelengths of 495 to 560 nm (green autofluorescence) and 560 to 620 nm (red autofluorescence) were simultaneously acquired on two additional nondescanned detectors and subtracted from the original SHG image. From the total of 170 images, 8 images were removed from analysis due to lack of tumor epithelial cells in the image and/or poor sample quality.

### Collagen density evaluation

Quantification of fibrillar collagen intensity was calculated with the use of Fiji Image Analysis software. Integrated collagen density values for each image were quantitated from the product of pixel intensity and positive area of the SHG collagen signal after subtraction of the background autofluorescence (green and red) signal from the original SHG image. The integrated density values for each region of interest within a histologic section (n = 5) were used to calculate the average collagen intensity for each tumor (n = 11 grade I/II and n = 9 grade III).

### TACS and boundary survey evaluation

SHG images were scored by seven evaluators for the presence or absence of tumor-stromal boundaries and, if present, the presence of TACS-1, -2 or -3 fibers. The evaluators possessed various training backgrounds and included two board-certified veterinary medical oncologists [KS, PS], two veterinary medical oncology residents [AC, JG], two board certified veterinary pathologists [ACD, LB], and one PhD [JM]. Corresponding regions were imaged and images were randomized using a random number generator. The same 7 evaluators scored all cases and the evaluators were blinded to the associated clinical case data. If a distinct tumor-stromal boundary could be identified, evaluators were asked if they could identify at least one or more fibers of each TACS. TACS-1 was defined as dense relaxed collagen fibers adjacent to tumor. TACS-2 was defined as a straightening of collagen fibers around the tumor leading to straight fibers that are aligned parallel to the tumor boundary. TACS-3 was defined as remodeling of the stroma with reorientation of collagen such that collagen fibers are bundled and aligned perpendicular to the tumor boundary [24, 38, 39]. The evaluators scored each image as positive (1) or negative (0) for the given variables (TACS-1, -2, -3 and presence of a boundary). Thus, 5 separate images from each tumor were evaluated by 7 different observers resulting in a total of 35 scores per tumor per variable. Data are presented as the average of the 35 scores for each variable for each tumor. Five additional tumors were identified, imaged and scored by all reviewers for presence or absence of boundary after statistical analysis revealed the initial set was not sufficiently powered.

### Computer-assisted collagen fiber analysis and TACS quantification

Using the SHG images described above, we analyzed collagen fiber orientation at defined regions of tumor-stromal boundary, as described previously [25, 40]. Tumor-stromal boundaries were identified and traced manually, and fibers at the tumor boundary were analyzed for TACS-2 and -3 using CurveAlign software (http://loci.wisc.edu/software/curvealign, version 3.0, beta 2). Collagen fibers were considered to be TACS-2^+^ if the angle between collagen fiber and tumor boundary were between 0 and 30 degrees or TACS-3^+^ if between 60 and 90 degrees. Images receiving an average boundary score of <0.5 by the seven reviewers (2 cases) were not analyzed via CurveAlign, as clear tumor-stromal boundaries are required for quantifying fiber angles. Additional collagen fiber characteristics (width, length, and straightness) were analyzed as described previously [41]. The CT-FIRE program was used to quantify individual collagen fiber parameters in every SHG image (http://loci.wisc.edu/software/ctfire, version 1.3, beta 2). For straightness, fibers were considered straight if the distance between fiber endpoints divided by the distance along the fiber is greater than the threshold value 0.92593 (1/1.08 = 0.92593) [23] and the data are presented as the percentage of all fibers that were straight.

### Statistical analysis

Unpaired two-tailed Mann-Whitney tests were used to determine the significance of differences between two groups. Data in box and whisker plots represent medians and ranges from maximum to minimum values. For Kaplan-Meier survival analysis, continuous variables were divided into two groups: Higher than the mean and lower than the mean of the variable being tested. Median OS and DFS were estimated using Kaplan-Meier survival analysis and the log-rank test was used to evaluate whether collagen intensity, presence of TACS-1, -2, -3, %TACS-2^+^ or -3^+^ fibers, the boundary score, or collagen fiber width, length, or straightness significantly impacted survival. Collagen intensity, fiber number, and fiber width were correlated to TACS-1 scores via Pearson product–moment correlation analysis (two-tailed). Study groups were compared using GraphPad Prism 5 statistical software (La Jolla, CA). p<0.05 was considered statistically significant.

Cox multivariable survival methods were used to determine which collagen signature variables (fiber length, fiber width, fiber straightness, integrated density, tumor stromal boundary, TACS-1, -2 and -3 score, and % TACS-2^+^ and -3^+^ fibers) were associated with OS or DFS. Each collagen signature variable was investigated in a well-specified base OS model and well specified base DFS model that contained the significant non-collagen signature variables (grade, stage, margins, OHE status, and lymphovascular invasion). Interactions among the main effects were investigated in each model. Variables with p<0.2 on univariate analysis (S2 Table) were considered in the multivariable model and variables were retained in final multivariable models if p<0.05. The assumption of proportional hazards was tested based on Schoenfeld residuals. These analyses were performed in Stata version 13 (StataCorp, College Station, TX).

## Results

**Fig 1:**
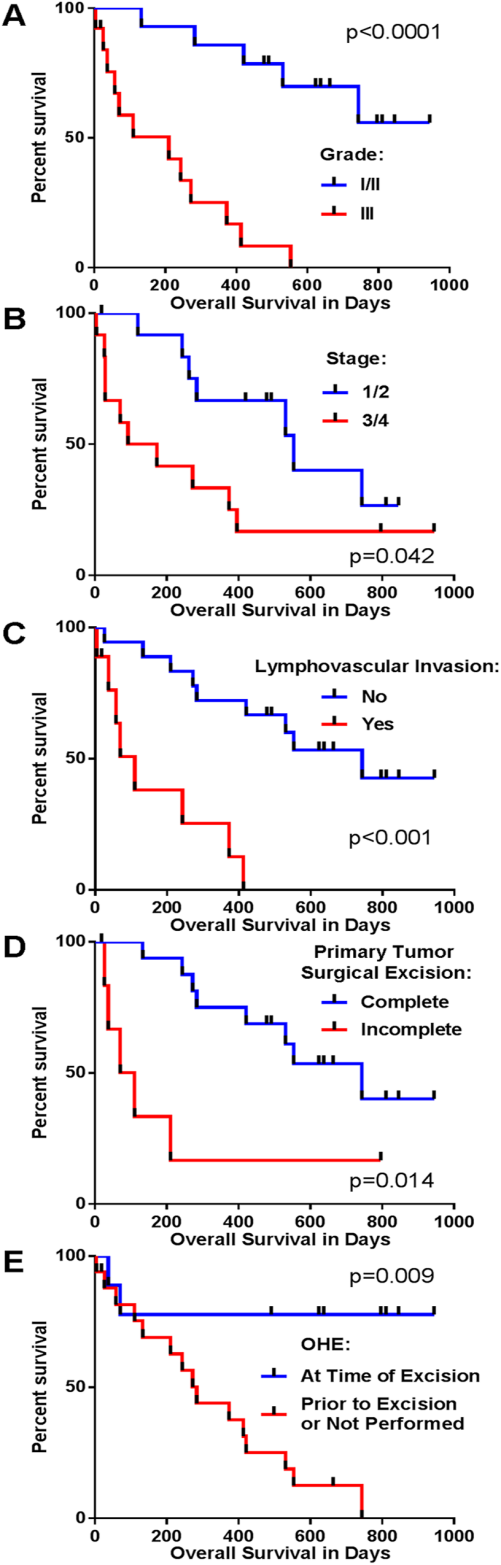
Canine mammary tumor clinical parameters predict outcome. Kaplan-Meier survival curves and Cox regression univariate analysis was used to evaluate whether clinical parameters significantly impacted survival. A. Grade: grade I/II vs. grade III mammary tumors (p<0.001, hazard ratio (HR) 10.106, 95%CI 3.071–33.254). B. Stage: Stage 1/2 vs. 3/4 mammary tumors (p = 0.042, HR 2.778, 95%CI 1.039–7.420). C. Lymphovascular Invasion: Evidence vs. No Evidence (p<0.001, HR 7.462, 95%CI 2.359–23.603). D. Excision Completeness: Complete vs. Incomplete (p = 0.014, HR 4.255, 95%CI 1.335–13.558). E. OHE: At time of excision vs. prior to excision or not performed (p = 0.009, HR 0.133, 95%CI 0.029–0.604).

To determine the ability of histologic grade to predict clinical outcome in our canine mammary tumors cohort, tumor grade (grade I/II vs. grade III) was compared with OS using Kaplan-Meier curves and Cox regression univariate analysis (Fig 1A). Consistent with previous studies, grade was a significant predictor of survival time (p<0.001). Compared to a median OS of 110 days for dogs with grade III tumors, dogs with grade I/II tumors had a median OS of 630 days. Although this shows that grade is generally a reliable predictor of survival, it is noteworthy that two out of 12 dogs (14%) with grade I tumors (stage 1–4 with outcome data) died of disease-related causes within a year after surgery (133 and 283 days). Similarly, 3 out of 13 grade III tumors (stage 1–4 with outcome data, 23%) in our cohort survived for more than three times the median OS of the remaining dogs in this group (373, 413, and 553 days beyond surgery). Given the small numbers of dogs with stage 4 disease at the time of diagnosis (N = 7), it was not surprising that dogs with stage ≤3 at diagnosis did not have a significantly different OS compared to those with stage 4 (median OS 450 (≤3) vs. 373 (4) days, p = 0.362). Dogs with stage 1 or 2 had a longer OS compared to those with stage 3 or 4 (Fig 1B, median OS 491 (1/2) vs. 160 (3/4) days, p = 0.042). Other clinical parameters tested included evidence of lymphovascular invasion on histopathology (Fig 1C, median OS 542 (No) vs. 70 (Yes) days, p<0.001), completeness of excision (Fig 1D, median OS 542 (Complete) vs. 70 (Incomplete) days, p = 0.014) and OHE performed at the time of primary tumor excision (Fig 1E, median OS 637 vs. 272 (prior to excision or not performed) days, p = 0.009. Cox regression univariate analyses for these data are also presented in S2 Table.

**Fig 2:**
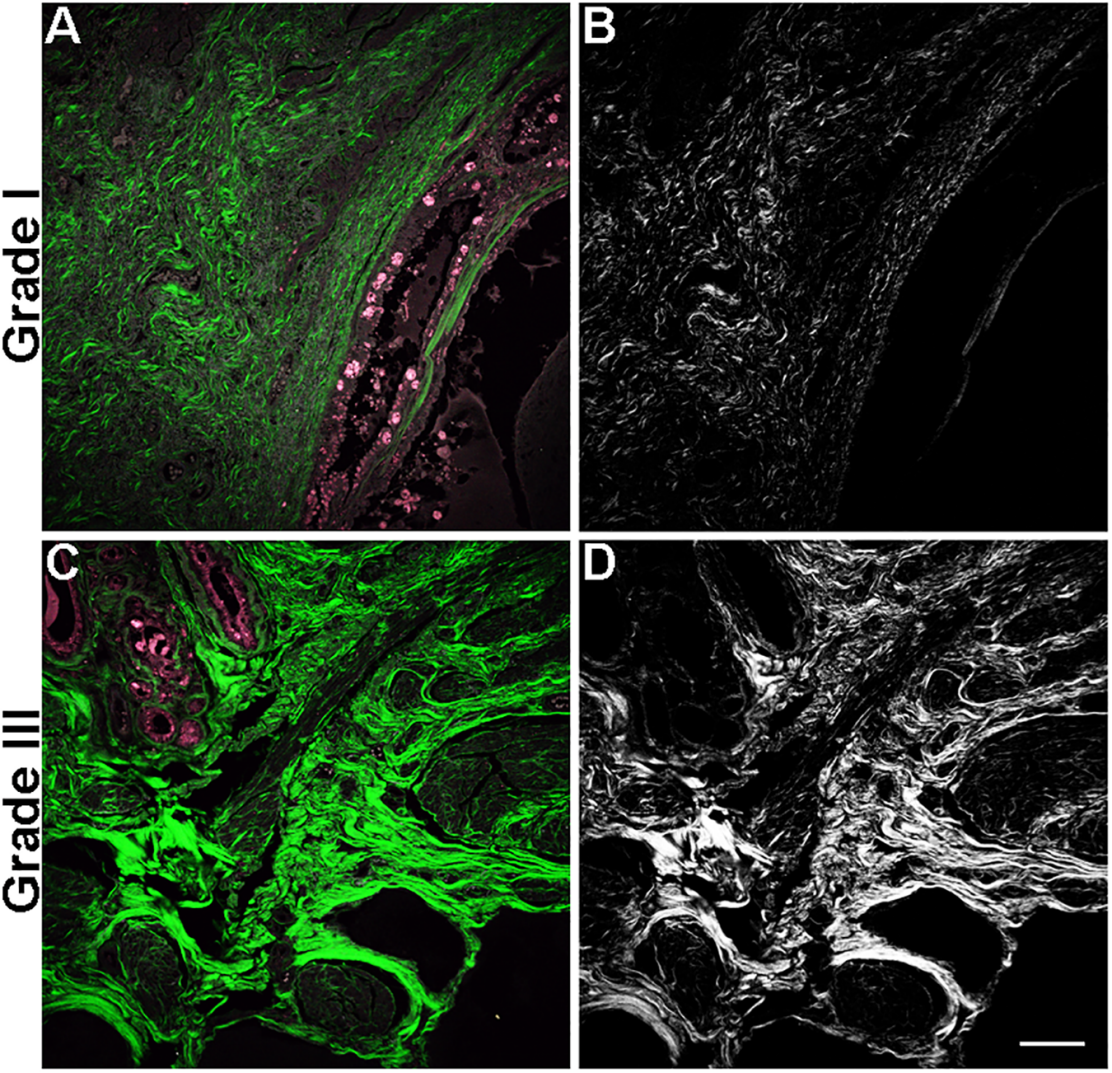
Fibrillar collagen in canine mammary tumors. Representative images of SHG signal (white, B and D; pseudocolored green A and C) and autofluorescence (pink, A and C) in low (grade I, A and B) and high-grade (grade III, C and D) mammary tumors. Scale bar = 50 μm.

**Fig 3:**
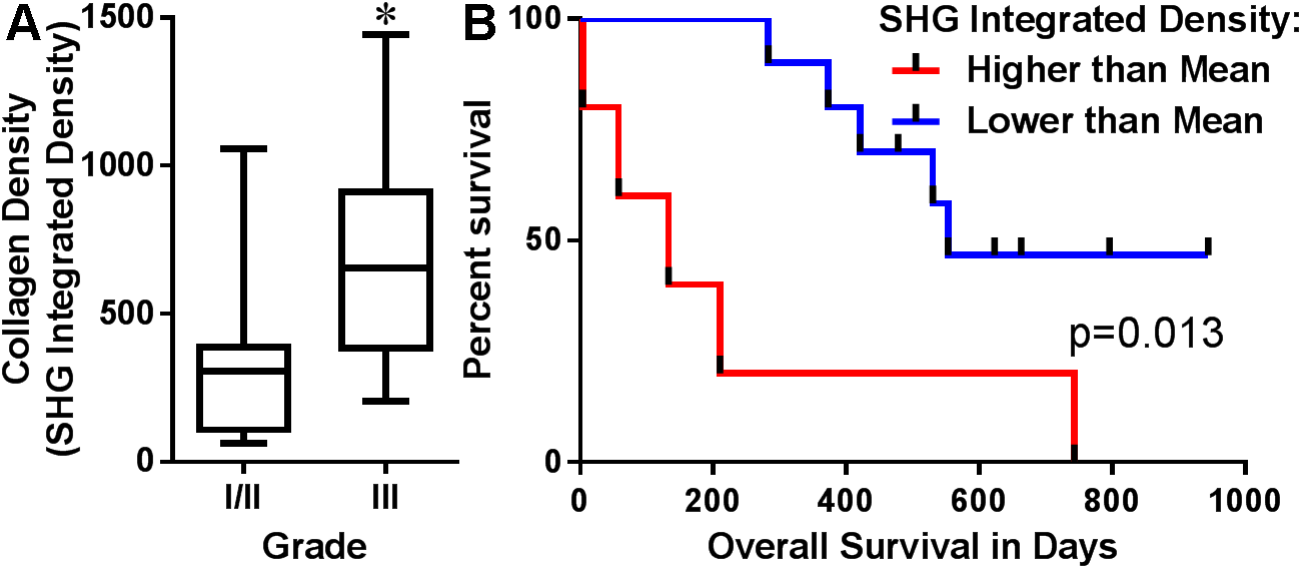
Collagen density predicts poor outcome in canine mammary tumors. Integrated density of collagen signal from SHG images was quantified using Fiji (Image J) software. Graph represents averages from 5 images per tumor from 11 grade I/II and 9 grade III mammary tumors. *p<0.05 via an unpaired Mann-Whitney test (A). Kaplan-Meier survival curve for 18 dogs with collagen integrated density higher or lower than the mean integrated density value (B). The log-rank test was used to evaluate whether the collagen density significantly impacted survival (p = 0.013, HR 4.099, 95%CI 0.860–19.520).

To determine if collagen density, fiber characteristics and TACS identified in routine histologic biopsy samples were associated with tumor grade and survival time of canine mammary carcinoma patients, SHG imaging was performed [42] in histologic sections of 34 canine mammary carcinoma biopsies (Fig 2). As an increase in collagen density has been shown to correlate with tumor invasiveness and poor clinical outcome in human breast cancer tissue [22, 43], we first quantified the intensity of the collagen fiber SHG signal in canine mammary gland tumor stroma in order to determine whether higher collagen density in canine mammary tumors was associated with grade and poor prognosis. Consistent with previous reports in human breast cancer [38], collagen density was increased in grade III tumors compared to that in grade I/II tumors (Fig 3A). A Kaplan-Meier survival curve showed a significant difference in OS between dogs with tumors that had higher than the mean collagen density (median OS = 133 days) compared to those dogs with tumors having a collagen density lower than the mean density (median OS = 553 days, p = 0.013 Fig 3B). However, in Cox multivariable regression models for OS, integrated density lost its prognostic value.

As recent data suggest that collagen organization is equally important as density in modulating breast cancer behavior [21, 23–25, 38, 39], we next examined TACS within SHG images using both an observer-based scoring method and quantitative data analysis software (CurveAlign). Recent studies have focused on collagen alignment perpendicular to the tumor boundary (TACS-3), which predicts poor breast cancer patient survival, independent of other standard clinical variables [20, 21, 23], and aggressive cancer behavior in murine models [24, 25, 39]. Seven evaluators scored SHG images for the presence of TACS-1, 2, and 3 (Table 1). There was no significant difference in survival for any of the three TACS via Kaplan-Meier and log-rank analysis. In Cox multivariable regression controlling for grade, stage, OHE status, and lymphovascular invasion, no TACS was significantly associated with OS.

**Table 1:** TACS in canine mammary tumor OS.

			Median OS (days)		Log-rank test		
			Lower than Mean	Higher than Mean	p-value	Hazard Ratio	95% Confidence Interval
Evaluators		TACS-1	553	530	0.304	1.789	0.550–5.821
		TACS-2	743	530	0.645	1.322	0.416–4.199
		TACS-3	463	743	0.457	0.655	0.206–2.084
Data Analysis Software		% TACS-2^+^ Fibers	407	421	0.469	0.697	0.261–1.859
		% TACS-3^+^ Fibers	421	472	0.369	1.564	0.553–4.427

TACS were scored by evaluators and measured with data analysis software (CurveAlign) from SHG images. Five images were analyzed for each tumor. Observers scored 22 tumors (from dogs with outcome data) and data represent the percent of images having at least one fiber characterized as TACS-1, 2, or 3. Collagen fiber angles in respect to the tumor-stromal boundary were analyzed from 26 tumors (from dogs with outcome data). The % TACS-2^+^ and -3^+^ fibers were determined by the angle (TACS-2 0–30°, TACS-3 60–90°) and data are presented as the percent of all fibers that were positive for TACS-2 or 3. Kaplan-Meier curves were generated using 2 groups: lower or higher than the mean.

To complement evaluator assessment of the presence of TACS in canine biopsies, we used data analysis software to measure the angle of the collagen fibers relative to the tumor-stromal boundary to determine the relative proportion of TACS-2^+^ and 3^+^ fibers as previously described [23, 38, 41]. To compare these methodologies, the %TACS-3^+^ fiber data (determined using the computer-analysis software) were compared between images that had scored negative or positive for TACS-3 via evaluators (S1 Fig). Concordance in our methodologies is supported by a significantly higher %TACS-3^+^ fibers in computer analyzed-images that were scored as TACS-3^+^ by the panel of evaluators. In Cox multivariable regression controlling for grade, stage, OHE status, and lymphovascular invasion, TACS-2^+^ and 3^+^ fibers were not significantly associated with OS.

**Fig 4:**
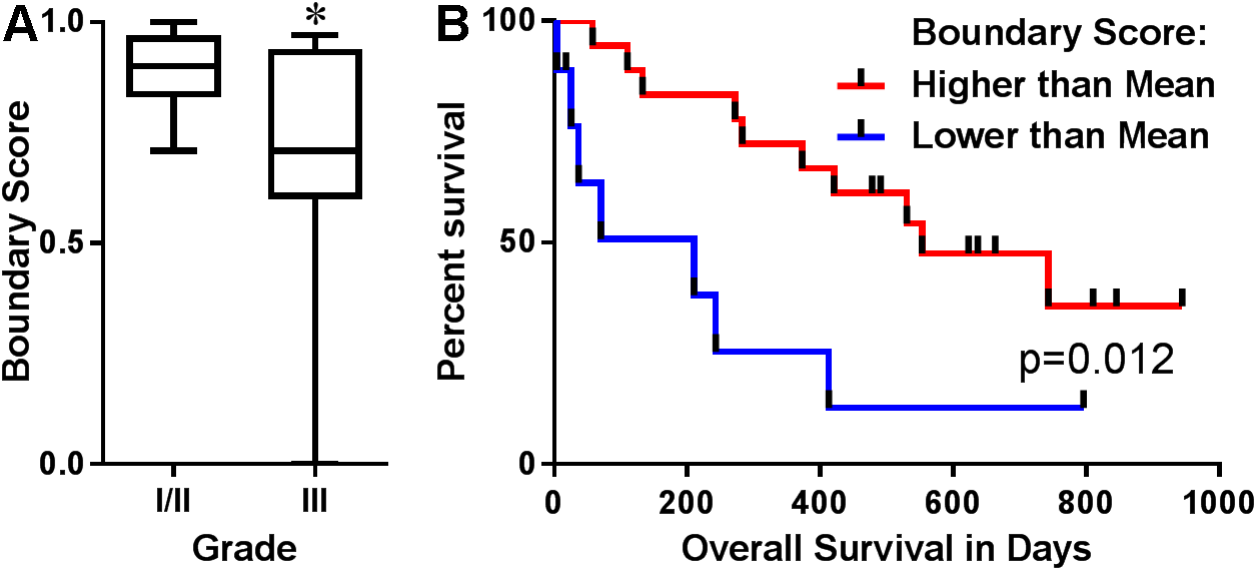
Absence of a tumor-stromal boundary predicts poor outcome in canine mammary tumors. A panel of 7 observers graded SHG images as having (1) or lacking (0) a tumor-stromal boundary. The boundary score for each tumor represents the average of the 7 evaluators’ scores and the 5 images per tumor. Boundary scores were compared between 15 grade I/II and 19 grade III tumors. *p<0.05 via a Mann-Whitney test (A). Kaplan-Meier survival curve for 27 dogs with boundary scores higher or lower than the mean boundary score (B). The log-rank test was used to evaluate whether the boundary score significantly impacted survival (p = 0.012, HR 0.316, 95%CI 0.092–1.087).

Based on these data and the observation that certain images lacked a clearly defined tumor-stromal boundary, we tested the secondary hypothesis that the absence of a clear tumor-stromal boundary, as assessed by the seven observers, was more common in high grade tumors and conversely that a defined tumor-stromal boundary was associated with prolonged survival times. In support of this hypothesis, grade I/II tumors were found to have significantly higher boundary scores, indicative of higher incidence of a clearly defined boundary, than grade III tumors (Fig 4A). There was also a significant difference in survival between tumors with a high boundary score and tumors with a low boundary score, with median OS of 553 and 210 days, respectively (p = 0.012; Fig 4B). In multivariable Cox regression analysis, controlling for stage, grade, OHE status, and lymphovascular invasion, the tumor-stromal boundary score was significantly associated with OS such that as the score increased, the risk of death decreased. The HR was 0.01 (p = 0.001) for dogs with disease stage <3 and low-grade tumors; and the HR was 0.02 (p = 0.01) for dogs with stage <3 and high-grade tumors. These results suggest that a lack of a tumor-stromal boundary represents a more aggressive tumor associated collagen signature on the continuum of progression and provides a plausible explanation for the lack of agreement between the prognostic value of TACS-3 in the dog and other species.

**Fig 5:**
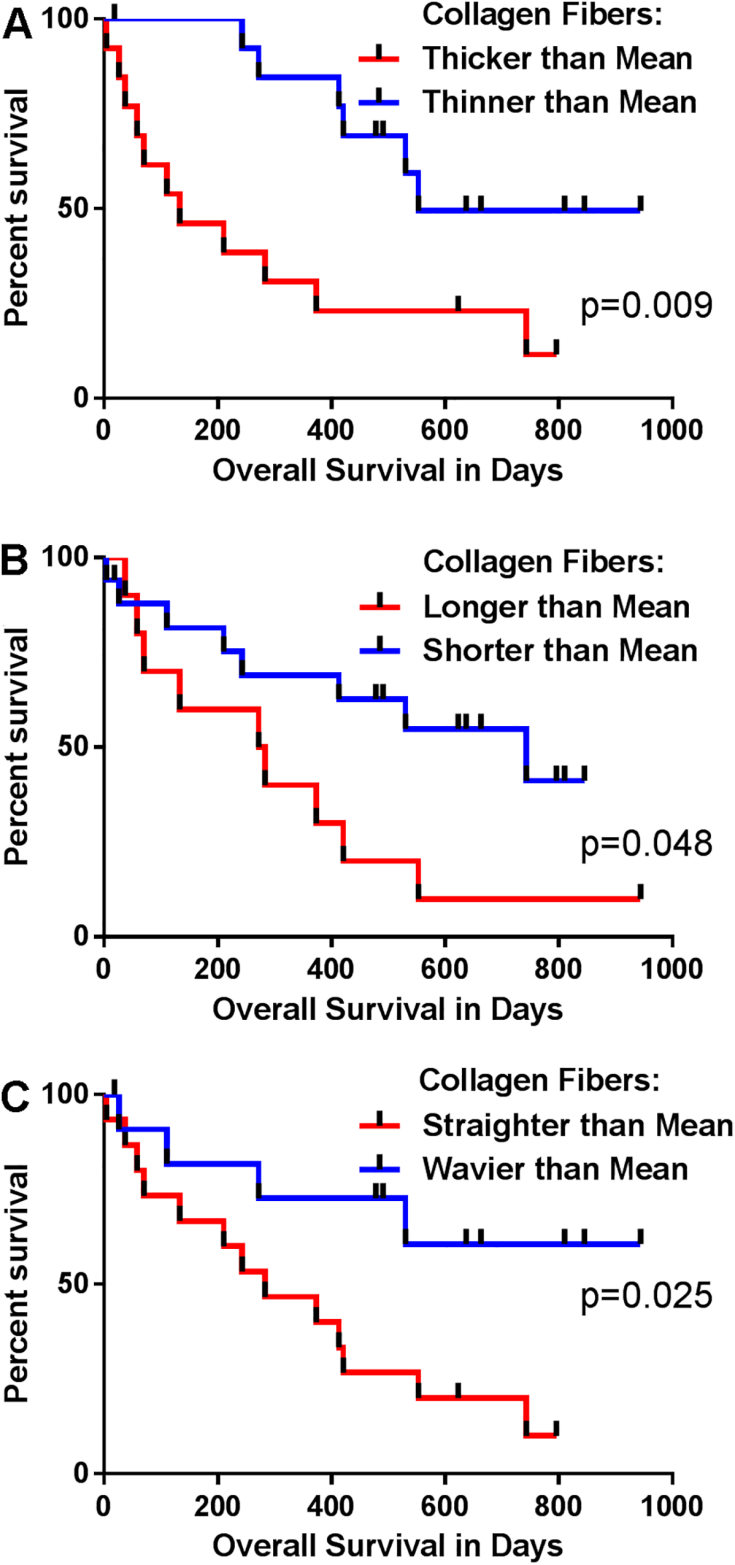
Collagen fiber characteristics in canine mammary carcinoma biopsies are associated with patient survival. Kaplan-Meier survival curve for 27 dogs, comparing lower and higher than the mean collagen fiber width (A; p = 0.009, HR 3.414, 95%CI 1.260–9.252) and length (B; p = 0.048, HR 2.501, 95%CI 0.895–6.990), as well as straightness (C; p = 0.025, HR 3.324, 95%CI 1.284–8.601), assessed by SHG analysis of histopathologic sections. The log-rank test was used to evaluate whether the collagen variable significantly impacted survival.

While quantifying collagen alignment at the tumor-stromal boundary has been shown to be useful in predicting human breast cancer survival [38], other collagen fiber characteristics also change during tumor progression and TME remodeling, and thus represent additional candidates for prognostic markers. Collagen fibers from canine mammary tumors were characterized on the basis of width, length and straightness using data analysis software (CT-FIRE). When survival times of dogs bearing tumors with collagen fibers thicker than the mean were compared to those thinner than the mean, increased width was found to negatively impact survival time (p = 0.009; Fig 5A). The median survival time was >4x longer for dogs with tumors containing collagen fibers thinner than the mean compared to those greater than the mean. Kaplan-Meier survival analysis showed that increasing fiber length also had a negative impact on survival (p = 0.048), as dogs with tumors with longer fibers had a mean survival of 278 days and dogs with tumors having shorter fibers had a mean survival of 743 days (Fig 5B). Finally, collagen fiber straightness was also found to negatively impact OS in Kaplan-Meier survival analysis (p = 0.025; Fig 5C). In the Cox multivariable regression analysis, controlling for stage, grade, OHE status, and lymphovascular invasion, fiber width, but not fiber length or straightness, was significantly associated with OS such that as fiber width increased, the risk of death increased. The HR was 7.13 (p = 0.02) for dogs with high grade tumors and stage 3 or 4 disease.

For all survival analyses, in addition to OS, DFS was analyzed. Overall, there were minimal changes in log-rank test results when evaluating DFS versus OS (S2 Table). Specifically, decreased boundary scores and increased collagen fiber width were associated with shorter DFS intervals, while collagen density was not significantly associated with DFS. In Cox multivariable regression models for DFS including tumor grade, lymphovascular invasion, and excisional completeness, the boundary score was significantly associated with DFS, such that as the boundary score increased, the risk of disease decreased for dogs that had tumors with clean margins. The HR for dogs with low-grade tumors and clean margins was 0.003 (p = 0.002) and the HR for dogs with high-grade tumors and clean margins was 0.002 (p = 0.011).

## Discussion

In this study, we examined the potential role of fibrillar collagen in modulating the biologic behavior of canine mammary gland tumors. We demonstrate that specific collagen signatures, revealed by SHG imaging in primary canine mammary carcinomas, are predictive of aggressive clinical outcomes. Specifically, we show that a lack of a defined tumor-stromal boundary and an increase in collagen fiber width are associated with poor outcomes even when tumor stage, grade, the effect of OHE performed at tumor excision, and evidence of lymphovascular invasion on histology are all considered. Although additional collagen fiber features (collagen density and collagen fiber length and straightness) were inversely correlated with patient OS, multivariate analyses revealed that these parameters were not associated with tumor progression when considered with clinical features such as stage, grade, OHE status, and lymphovascular invasion. It should be noted that even if these parameters fail to provide prognostic information beyond currently used clinical and histopathological parameters, targeting drivers of these tumor-permissive collagen features may improve on existing therapeutic strategies for canine mammary gland carcinoma. To the authors’ knowledge, this is the first study to investigate collagen density, organization and fiber characteristics as biologic drivers of canine mammary tumors. Collectively, our findings support further investigation of collagen biomarkers for prognostication of canine mammary tumors and suggest that stromal targeting may improve therapeutic success in these dogs.

Similar to other reports examining the prognostic value of grading on canine mammary carcinoma, tumor grade significantly affected outcome in our study (Fig 1). Previous studies have reported that 3–9% (1/29 and 2/23) of dogs with grade I tumors compared to 59–62% (10/17 and 16/26) of dogs with grade III tumors developed metastasis [10, 11]. In this study, 14% (2/14) of the dogs with grade I/II tumors and 53% (8/15) grade III tumors with available outcome data developed documented metastatic disease during the follow-up period. This metastatic rate is similar to that recently reported in women with metastatic breast cancer, where 14% of patients diagnosed with primary metastatic disease and an additional 7% of those that develop metastatic disease subsequent to treatment have grade I tumors [44]. Based on these findings, it is apparent that use of histologic grade, even when combined with other prognostic information including stage and histologic features, may lead to the over-treatment of a subpopulation of dogs with grade III tumors that would never have developed metastatic disease and the under-treatment of a significant number of dogs (up to 22%) with metastatic grade I tumors. Moreover, dogs with grade II tumors have an even more diverse outcome, with 16–36% (3/19 and 10/28) developing metastatic disease [10, 11], making clinical therapeutic recommendations even more challenging and the need for superior prognostic tools greater in this particular group. Future studies to define prognostic markers in this patient population are on-going and may provide a much needed clinical tool to determine necessity of adjuvant therapy post-surgery in dogs with mammary carcinomas.

Elevated mammographic density, which correlates with increased collagen content and alignment, is a known risk factor for the development of breast cancer [22] and metastasis to the lymph node [45]. Similar to human breast cancer, collagen density in canine mammary carcinoma biopsies was inversely related to patient survival. However, controlling for stage, grade, OHE status and lymphovascular invasion, collagen density was not significantly associated with OS. Despite the fact that density could not be used to improve prognostication beyond currently used markers, our data suggest that therapeutic strategies that suppress desmoplastic responses, particularly tumor-permissive collagen signatures may improve survival times. Numerous recent studies have revealed that organization and stiffness of the collagen matrix are equally as important as density in mediating tumor growth and invasion [17–19, 21, 38, 43], and support the ability of collagen to play both tumor-permissive and tumor-restrictive roles. Based on accumulating evidence that unique collagen signatures can predict clinical outcome in a variety of cancer types, including breast cancer [25, 38, 40], we applied these analyses to our canine mammary carcinoma biopsy samples. Although TACS-3 has been used to predict poor survival in human breast cancer patients [38], TACS-2 and -3 were not predictive of survival in the dog. This finding may be explained by our tumor-stromal boundary results but it is also likely that our very small sample size compared to much larger sample sizes used to study TACS in women with breast cancer [20, 23] may have limited our ability to detect prognostic significance of TACS-2 and -3 in the dog.

Investigating the presence of a tumor-stromal boundary and its impact on outcome has not been previously reported in mammary cancer. Tumors without a tumor-stromal boundary had collagen throughout the primary tumor, and SHG images showed collagen fibers intermingled among individualized or small groups of tumor epithelial cells (Fig 2). As TACS-1, 2, and 3 have been referred to as progressively aggressive collagen reorganization patterns, it is possible that the lack of a defined tumor-stromal boundary represents further progression along a continuum of collagen reorganization that has not been previously described. By definition, a tumor-stromal boundary is required to assign a TACS phenotype. Therefore, in our cohort of patients with the worst outcome, it is possible that the most aggressive tumors contained fibers that had progressed beyond TACS-3 to tumors lacking a clearly defined tumor-stromal boundary, or never possessed tumor-restrictive collagen signatures, and thus have no increase in TACS-3 that we could detect without a clear tumor-stromal boundary. This is plausibly accentuated in the dog compared to other species due to the fact that disease-bearing dogs tend to present for treatment at later stages than humans and mouse models tend to be less heterogeneous [32].

In addition to collagen density and organization, recent studies have also focused on individual collagen fiber characteristics as both drivers of, and evidence for, the dynamic reciprocity that exists between both neoplastic and stromal cells and collagens in the TME. Elegant computational analyses have been developed that allow quantitative assessments of these collagen characteristics from routine biopsy specimens following SHG imaging and correlation with aggressive tumor behaviors [41]. Collagen fiber thickness *in vitro* was found to increase human cancer cell invasiveness [46], while *in vivo*, straighter, longer, and wider fibers were found in pancreatic cancer compared to normal pancreas [47], and increased collagen fiber length correlates with poor patient survival in head & neck, esophageal and colorectal cancers [48]. However, these collagen characteristics have not been shown to uniformly drive aggressive cancer behavior as decreased invasion was seen in glioblastoma biopsies within regions containing long, thick, and straight fibers [49]. Although we found that, on univariate analysis, collagen fiber width, length and straightness were significantly associated with OS in canine mammary carcinoma patients, only collagen fiber width was associated with OS when considered with stage, grade, OHE status, and lymphovascular invasion in multivariable analysis. Interestingly, only collagen fiber width was associated with DFS via univariate analysis (S3 Table). It is possible that limitations in our ability to accurately document disease progression in our PVDL population that were monitored less consistently post-operatively limited our ability to determine an association between collagen fiber length and straightness, or other, parameters. In addition, the small sample size in our study may have further contributed to our inability to identify such associations between specific collagen signatures or other parameters such as stage (stages 1–3 vs 4) with respect to outcome (type II error). Nonetheless, it appears the roles that individual collagen characteristics play in modulating cancer behavior are dependent on the type of cancer and animal model. This highlights the need for further studies to define collagen parameters that are predictive of outcome in tumor models and in patient populations.

Finally, as this work confirms similarities between the mammary TME of women and dogs, it may have important implications for human breast cancer. This is particularly important as murine breast cancer models, especially xenograft models [50], may fail to elicit a robust desmoplastic reaction similar to that seen in human and canine patients. As such, this work supports the use of the dog as a complementary translational model to help define mechanisms that drive the formation of tumor-inciting collagen signatures and to aid in the assessment of the efficacy and safety of therapeutic targets that disrupt or reverse their formation. Although murine models play a key role in breast cancer research, the dog has additional advantages as a model for human breast cancer. Most notably, spontaneous canine mammary tumors develop in the context of an intact immune system and share significant similarities with human breast cancer with respect to clinical presentation, genetics, molecular marker expression, hormone dependency and disease progression [8, 13, 16, 34, 51–54]. Furthermore, client-owned dogs are a genetically diverse population, are of large body size, share similar carcinogenic environmental risk factors, and undergo similar oncologic diagnostics and therapeutics [55, 56]. This heterogeneity at both the patient and tumor levels more accurately models human patients. Our study, which is the first to define prognostic collagen signatures in canine mammary tumors, makes positive contributions to both veterinary and human breast cancer research and supports a one-health approach.

## Supporting information

S1 Fig Agreement in TACS-3 evaluation of canine mammary tumors by different methods. The percentage of (%) TACS-3^+^ fibers was determined using computer-analysis software (CurveAlign) and was compared between images that had scored negative or positive for TACS-3 via evaluators. **p<0.01 via an unpaired Mann-Whitney test.
(TIF)

S2 Fig Evaluator TACS-1 scores agree with collagen fiber analysis. Scatter plots showing comparison of the evaluator scores for TACS-1 to the integrated density (A) and width (B) of the collagen fibers or to total fiber number per image (C). Correlations were evaluated using Pearson product–moment correlation analysis. TACS-1 had positive correlations with collagen fiber integrated density, width, and total fiber number, indicating that the computer analyzed collagen density and CT-FIRE quantification of collagen density (influenced by both collagen width and fiber number) was in agreement with the evaluators’ assessment of TACS-1.
(TIF)

S1 Table Clinical diagnostic, treatment, and outcome data. Clinical variables of dogs enrolled in this study. DFS, Disease-Free Survival; OS, Overall Survival; OHE, ovariohysterectomy; NA, not able to assess (the entire tumor was not submitted for histological review); *, not included in outcome analysis.
(DOCX)

S2 Table Univariate analysis. The effects of clinical variables on overall survival and disease-free survival. OHE, ovariohysterectomy.
(DOCX)

S3 Table Collagen variables effects on overall survival and disease-free survival. Collagen characteristics were analyzed to determine any impact on OS or DFS via Kaplan-Meier survival curves and log-rank tests. OS, Overall Survival; DFS, Disease-Free Survival; CI, Confidence Interval; *p < 0.05; **p <0.01.
(DOCX)
