# Supplementary figures and images for: Identification of prognostic collagen signatures and potential therapeutic stromal targets in canine mammary gland carcinoma
Source: PLoS One. 2017 Jul 6;12(7):e0180448. doi: 10.1371/journal.pone.0180448 (PMC5500345; doi:10.1371/journal.pone.0180448)

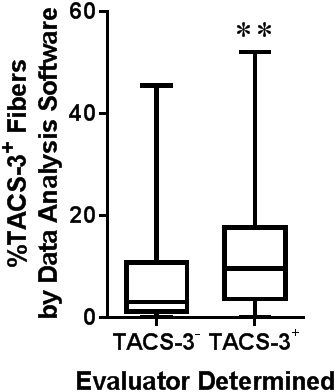

Supplement: S1 Fig — The percentage of (%) TACS-3+ fibers was determined using computer-analysis software (CurveAlign) and was compared between images that had scored negative or positive for TACS-3 via evaluators. **p<0.01 via an unpaired Mann-Whitney test. (TIF) [file pone.0180448.s001.tif]

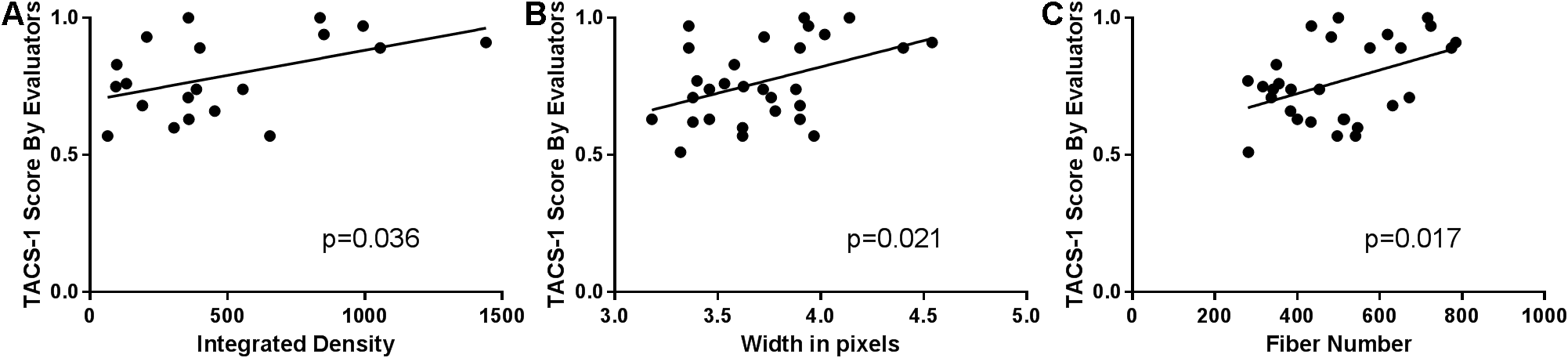

Supplement: S2 Fig — Scatter plots showing comparison of the evaluator scores for TACS-1 to the integrated density (A) and width (B) of the collagen fibers or to total fiber number per image (C). Correlations were evaluated using Pearson product–moment correlation analysis. TACS-1 had positive correlations with collagen fiber integrated density, width, and total fiber number, indicating that the computer analyzed collagen density and CT-FIRE quantification of collagen density (influenced by both collagen width and fiber number) was in agreement with the evaluators’ assessment of TACS-1. (TIF) [file pone.0180448.s002.tif]
