# Supplementary material for: Identification of prognostic collagen signatures and potential therapeutic stromal targets in canine mammary gland carcinoma
Source: PLoS One. 2017 Jul 6;12(7):e0180448. doi: 10.1371/journal.pone.0180448 (PMC5500345; doi:10.1371/journal.pone.0180448)
Supplement: S1 Table — Clinical variables of dogs enrolled in this study. DFS, Disease-Free Survival; OS, Overall Survival; OHE, ovariohysterectomy; NA, not able to assess (the entire tumor was not submitted for histological review); *, not included in outcome analysis. (DOCX) [file pone.0180448.s003.docx]

**S1 Table. Clinical diagnostic, treatment, and outcome data.**

|  | DFS (Days) | Tumor related event  PT=local recurrence or new primary tumor; DM=distant metastasis | OS (Days) | Reason for Censorship | Stage  (based on available data) | Grade | Evidence for lymphovascular invasion on histopathology  Yes (Y) or No (N) | Completeness of excision | OHE status  (pre-/post-operative)  F=female intact  FS=female spayed  U=undocumented |
| --- | --- | --- | --- | --- | --- | --- | --- | --- | --- |
| PVSCMTP1 | 796 |  | 796 | Alive | 3 | I | N | Incomplete | F/FS |
| PVSCMTP 2 | 944 |  | 944 | Alive | 4 | II | N | Complete | F/FS |
| PVSCMTP 3 | 491 |  | 491 | Alive | 1 | I | N | Complete | F/FS |
| PVSCMTP 4 | 810 |  | 810 | Alive | 1 | I | N | Complete | F/FS |
| PVSCMTP 5 | 419 | DM | 637 | Lost to follow up | 2 | I | N | Complete | F/FS |
| PVSCMTP 6 | 845 |  | 845 | Alive | 1 | I | N | Complete | F/FS |
| NULS1 | 243 | DM | 243 | Not Censored | 2 | III | Y | Complete | F/F |
| NULS2 | 26 | DM | 26 | Not Censored | 4 | III | N | Incomplete | F/F |
| NULS3 | 28 | DM | 37 | Not Censored | 4 | III | Y | Incomplete | F/FS |
| NULS4 | 92 | DM | 110 | Not Censored | 3 | III | Y | Incomplete | F/F |
| NULS5 | 70 | DM | 70 | Not Censored | 3 | III | Y | Incomplete | F/FS |
| PVDL1 | 18 | PT | 18 | Lost to follow up | 1 | III | Y | Incomplete | F/F |
| PVDL2* | 0 | DM | 10 | Not Censored | 5 | III | Y | Complete | FS/FS |
| PVDL3 | 119 | PT/DM | 133 | Not Censored | 1 | I | N | Complete | FS/FS |
| PVDL4 | 28 | DM | 58 | Not Censored | 3 | III | Y | NA | F/F |
| PVDL5 | 395 | PT | 413 | Not Censored | 4 | III | Y | NA | FS/FS |
| PVDL6 | 272 |  | 272 | Not Censored | 3 | III | N | Complete | FS/FS |
| PVDL7 | 530 |  | 530 | Not Censored | 1 | I | N | Complete | FS/FS |
| PVDL8 | 553 |  | 553 | Not Censored | 1 | III | N | Complete | FS/FS |
| PVDL9 | 177 | PT | 663 | Lost to follow up | ≤3 | I | N | Complete | FS/FS |
| PVDL10 | 478 |  | 478 | Lost to follow up | 1 | I | N | Complete | U |
| PVDL11 | 373 |  | 373 | Not Censored | 4 | III | Y | NA | FS/FS |
| PVDL12* | 0 | DM | 62 | Not Censored | 5 | III | Y | Complete | FS/FS |
| PVDL13 | 172 | PT | 210 | Not Censored | 3 | III | N | Incomplete | FS/FS |
| PVDL14 | 623 | PT | 623 | Lost to follow up | ≤3 | I | N | Complete | F/FS |
| PVDL15 | 262 | PT | 421 | Not Censored | 1 | II | N | Complete | FS/FS |
| PVDL16 | 283 | PT | 283 | Not Censored | 1 | I | N | Complete | F/F |
| PVDL17 | 4 |  | 4 | Not Censored | 3 | III | Y | NA | F/F |
| PVDL18 | 743 |  | 743 | Not Censored | 1 | I | N | Complete | FS/FS |
| **Additional biopsy specimens used to analyze relationship between collagen signatures and grade only (No clinical outcome data available)** | | | | | | | | | |
| PVDL19* |  |  |  |  | 4 | III | Y | NA | FS/FS |
| PVDL20* |  |  |  |  | 3 | III | N | Incomplete | FS/FS |
| PVDL21* |  |  |  |  | ≤3 | I | N | Complete | F/F |
| PVDL22* |  |  |  |  | 3 | III | Y | Complete | F/F |
| PVDL23* |  |  |  |  | 4 | III | Y | Incomplete | F/FS |

Clinical variables of dogs enrolled in this study.

DFS, Disease-Free Survival; OS, Overall Survival; OHE, ovariohysterectomy; NA, not able to assess (the entire tumor was not submitted for histological review); *, not included in outcome analysis.
