# Supplementary material for: Identification of prognostic collagen signatures and potential therapeutic stromal targets in canine mammary gland carcinoma
Source: PLoS One. 2017 Jul 6;12(7):e0180448. doi: 10.1371/journal.pone.0180448 (PMC5500345; doi:10.1371/journal.pone.0180448)
Supplement: S2 Table — The effects of clinical variables on overall survival and disease-free survival. OHE, ovariohysterectomy. (DOCX) [file pone.0180448.s004.docx]

**S2 Table. Univariate Analysis.**

|  | Overall survival | | | Disease-free survival | | |
| --- | --- | --- | --- | --- | --- | --- |
|  | Hazard ratio | 95% Confidence Interval | p-value | Hazard ratio | 95% Confidence Interval | p-value |
| Stage | 2.778 | 1.040-7.420 | 0.042 | 2.087 | 0.689-6.320 | 0.193 |
| Grade | 10.106 | 3.071-33.254 | <0.001 | 4.401 | 1.415-13.690 | 0.010 |
| Lymphovascular invasion | 7.462 | 2.359-23.603 | <0.001 | 5.024 | 1.642-15.373 | 0.005 |
| Completeness of surgical excision | 4.255 | 1.335-13.558 | 0.014 | 5.521 | 1.763-17.288 | 0.003 |
| OHE | 0.133 | 0.0294-0.604 | 0.009 | 0.379 | 0.114-1.258 | 0.113 |

The effects of clinical variables on overall survival and disease-free survival.

OHE, ovariohysterectomy
