# Supplementary material for: Identification of prognostic collagen signatures and potential therapeutic stromal targets in canine mammary gland carcinoma
Source: PLoS One. 2017 Jul 6;12(7):e0180448. doi: 10.1371/journal.pone.0180448 (PMC5500345; doi:10.1371/journal.pone.0180448)
Supplement: S3 Table — Collagen characteristics were analyzed to determine any impact on OS or DFS via Kaplan-Meier survival curves and log-rank tests. OS, Overall Survival; DFS, Disease-Free Survival; CI, Confidence Interval; *p < 0.05; **p <0.01. (DOCX) [file pone.0180448.s005.docx]

**S3 Table. Collagen Variables effects on Overall Survival and Disease-Free Survival.**

|  |  | OS | | | DFS | | |
| --- | --- | --- | --- | --- | --- | --- | --- |
| Variable | Comparison | p-value | Hazard Ratio | 95% CI | p-value | Hazard Ratio | 95% CI |
| Intensity | Higher vs Lower than Mean | 0.013* | 4.099 | 0.860-19.520 | 0.104 | 3.182 | 0.483-20.970 |
| TACS-1 | Higher vs Lower than Mean | 0.304 | 1.789 | 0.550-5.821 | 0.431 | 1.637 | 0.456-5.873 |
| TACS-2 | Higher vs Lower than Mean | 0.645 | 1.322 | 0.416-4.199 | 0.269 | 0.503 | 0.144-1.760 |
| TACS-3 | Higher vs Lower than Mean | 0.457 | 0.655 | 0.206-2.084 | 0.520 | 0.668 | 0.189-2.366 |
| %TACS-2 | Higher vs Lower than Mean | 0.469 | 0.697 | 0.261-1.859 | 0.206 | 0.508 | 0.176-1.432 |
| %TACS-3 | Higher vs Lower than Mean | 0.369 | 1.564 | 0.553-4.427 | 0.328 | 1.677 | 0.540-5.209 |
| Boundary | Higher vs Lower than Mean | 0.012* | 0.316 | 0.092-1.087 | 0.005** | 0.266 | 0.073-0.969 |
| Width | Higher vs Lower than Mean | 0.009* | 3.414 | 1.260-9.252 | 0.044* | 2.729 | 0.948-7.861 |
| Length | Higher vs Lower than Mean | 0.048 | 2.501 | 0.895-6.990 | 0.381 | 1.565 | 0.518-4.729 |
| Straightness | Higher vs Lower than Mean | 0.025 | 3.824 | 1.284-8.601 | 0.141 | 2.167 | 0.787-5.967 |

Collagen characteristics were analyzed to determine any impact on OS or DFS via Kaplan-Meier survival curves and log-rank tests.

OS, Overall Survival; DFS, Disease-Free Survival; CI, Confidence Interval; *p < 0.05; **p <0.01.
